# Retrospective evaluation of the incidental finding of 403 papillary thyroid microcarcinomas in 2466 patients undergoing thyroid surgery for presumed benign thyroid disease

**DOI:** 10.1186/s12885-015-1352-4

**Published:** 2015-04-30

**Authors:** Nikola Slijepcevic, Vladan Zivaljevic, Jelena Marinkovic, Sandra Sipetic, Aleksandar Diklic, Ivan Paunovic

**Affiliations:** 1 Centre for endocrine surgery, Clinical Centre of Serbia, Koste Todorovica 8, Belgrade, 11000 Serbia; 2 School of Medicine, University of Belgrade, Dr Subotica 8, Belgrade, 11000 Serbia; 3 Institute of Medical Statistics and Informatics, School of Medicine, University of Belgrade, Dr Subotica 8, Belgrade, Serbia; 4 Institute of Epidemiology, School of Medicine, University of Belgrade, Visegradska 26a, Belgrade, 11000 Serbia

**Keywords:** Benign thyroid disease, Thyroid surgery, Papillary thyroid microcarcinoma, Incidence, Predictors

## Abstract

**Background:**

The aim of our study was to investigate the incidence of papillary thyroid microcarcinoma (PTMC) in patients operated for benign thyroid diseases (BTD) and its relation to age, sex, extent of surgery and type of BTD.

**Methods:**

Retrospective study of 2466 patients who underwent thyroid surgery for BTD from 2008 to 2013. To determine independent predictors for PTMC we used three separate multivariate logistic regression models (MLR).

**Results:**

There were 2128 (86.3%) females and 338 (13.7%) males. PTMC was diagnosed in 345 (16.2%) females and 58 (17.2%) males. Age ranged from 14 to 85 years (mean 54 years). Sex and age were not related to the incidence of PTMC. The overall incidence of PTMC was 16.3%. The highest incidence was in Hashimoto thyroiditis (22.7%, χ^2^ = 10.80, p < 0.001); and in patients with total/near-total thyroidectomy (17.7%, χ^2^ = 7.05, p < 0.008). The lowest incidence (6.6%, χ^2^ = 9.96, p < 0.001) was in a solitary hyperfunctional thyroid nodule (SHTN). According to MLR, Hashimoto thyroiditis (OR 1.54, 95% CI 1.15-2.05, p < 0.003) and SHTN (OR 0.43, 95% CI 0.21-0.87, p < 0.019) are independent predictors. Since the extent of surgery was an independent predictor (OR 1.45, 95% CI 1.10-1.92, p = 0.009) for all BTD, and sex and age were not; when the MLR model was adjusted for them, Graves disease (OR 0.72, 95% CI 0.53-0.99, p < 0.041) also proved to be an independent predictor.

**Conclusions:**

Sex and age are not statistically related to the incidence of PTMC in BTD. The incidence of PTMC is higher in Hashimoto thyroiditis and patients with total/near-total thyroidectomy; and lower in patients with a SHTN and Graves disease.

## Background

Thyroid cancer (TC) is considered the most common malignancy of the endocrine system, with an incidence that ranges from 1 to 8 per 100,000 [1]. Over the past few decades, TC incidence has dramatically increased, and is considered, in some parts of the world, the second, and even the first most common cancer in women [2,3]. This is even more intriguing when we take into account that the overall cancer incidence rates for other localizations have mainly decreased for both women and men [4]. This remarkable increase of TC incidence is associated mainly with well-differentiated TC, above all papillary thyroid cancer (PTC). PTC constitutes more than 70% of TC [5]. This increase in the incidence of TC is mainly recognized as an increased detection of papillary thyroid microcarcinoma (PTMC) [6]. PTMC is defined as tumours of less than or equal to 10mm in diameter [7]. PTMC can be non-incidental or incidental. Non-incidental PTMC is usually diagnosed on the basis of fine-needle aspiration biopsy (FNAB), local or distant metastasis. Incidental PTMC is most commonly discovered on definitive paraffin section examination following thyroid surgery for benign thyroid disease (BTD). PTMC has a different incidence rate compared to clinically evident PTC. There is no universally accepted approach to PTMC and treatment ranges from observation, hemithyroidectomy, total thyroidectomy and total thyroidectomy with central lymph node dissection followed by radioactive iodine treatment [8,9]. The aim of our study was to investigate the incidence of PTMC in patients operated for BTD, establish whether its incidence is related to age, sex, extent of surgery and type of BTD and identify potential independent positive and negative predictors of PTMC.

## Methods

A retrospective study was conducted at a high volume specialized endocrine surgery unit of a tertiary referral university hospital. The study was approved by the ethic committee of the tertiary referral university hospital. Data were gathered from the database of all consecutive patients who underwent thyroid surgery for BTD in a five-year period (May 2008 to May 2013). All of these patients, during the process of making the diagnosis of BTD, undergo a standard diagnostic workup which includes laboratory tests, ultrasound examinations, and in selected cases scintigraphy, FNAB (fine needle aspiration biopsy) and a chest X-ray. All patients with clearly palpable non-hyperfunctional thyroid nodules greater than 10mm and with a normal value of calcitonin, undergo FNAB. We routinely test calcitonin in all of our patients with thyroid nodules of any size, thus eliminating the possibility of overlooking medullary thyroid cancer. We usually do not perform FNAB for nodules of size less than 10mm, since ultrasound guided FNAB is not routinely used in our healthcare setting.

All patients that underwent thyroid surgery for BTD, with or without an incidentally discovered PTMC on definitive paraffin section examination, were included in the study. All our pathohistological examinations are performed by three experienced endocrine pathologists with more than 20 years of working experience in this field. The protocol for pathohistological examination follows well established standards. A standard hematoxylin and eosin staining protocol for examining surgical specimens is used, whereas immunohistochemistry is used in selected cases. If the diagnosis is uncertain, the final diagnosis is based on the consensus of two pathologists. Patients with non-incidental PTMC were excluded from the study, as were all patients that had, apart from BTD, a TC greater than 10mm. All patients that had medullary TC or non-papillary TC of any size were also excluded from the study. The studied group consisted of 2466 patients with BTD of which 403 patients had an incidentally discovered PTMC on definitive paraffin section examination. In the studied group of patients with BTD, the most common indications for surgery were hyperthyroidism, a solitary nodule and thyroid enlargement with compressive symptoms (dysphagia, dyspnoea, hoarseness).

We analysed sex, age, type of BTD and extent of surgery in relation to the incidence of PTMC. According to type of BTD, patients were classified into the following groups: patients with multinodular goitre (MNG), Hashimoto thyroiditis, Graves’s disease, Plummer’s disease, solitary hyperfunctional thyroid nodule (SHTN) and benign tumours (which included colloid adenoma, follicular adenoma, Hurthle-cell adenoma and thyroid cysts). Within the group of benign thyroid tumours we did not find any statistical differences in relation to PTMC. Thus we formed one group - benign tumours. The extent of surgery was classified into two groups: HT group – less than total thyroidectomy (Dunhill procedure, hemithyroidectomy with isthmectomy and other less radical procedures) and TT group – total thyroidectomy (near total or total thyroidectomy).

Regarding age, a ten-year age group interval was used because of the small numbers that a five-year age group would produce resulting in unstable rates. For the same reason the age groups <20 and 70+ were formed. The statistical relationship between incidence of PTMC and sex, age, type of BTD and extent of surgery were first tested with Pearson's Chi-Square test and Fisher's exact test, with a two-sided value of p. To determine independent predictors for PTMC we used three separate multivariate logistic regression models (MLR). The first model tested all variables and their relation to PTMC. All variables that were statistically related to PTMC in univariate logistic regression (ULR) at the level of significance of p < 0.1, were included in the MRL model. In the second model we tested all variables and their relation to the extent of surgery. Here, also, all variables that were statistically related to the extent of surgery in ULR at the level of significance of p < 0.1, were included in the MRL model. Finally, in the third model we performed MLR for each type of BTD in relation to PTMC but adjusted for sex, age and extent of surgery. This MLR model was adjusted for sex, age and extent of surgery, since sex and age were not statistically significantly related to PTMC; and extent of surgery was statistically significantly related to PTMC and type of BTD for each BTD, except for MNG. SPSS version 16.0.2 (SPSS Inc., Chicago, Illinois, USA) was used to perform the statistical analysis.

## Results

The incidence of PTMC in relation to sex, age group, type of BTD and extent of surgery are presented in Table 1. The overall incidence of PTMC in BTD was 16.3%. In our studied group of patients with BTD (2128 females and 338 males) women outnumbered men sixfold (6.3:1). In our group of patients with BTD and PTMC (345 females and 58 males), a similar ratio was noted (5.9:1). The incidence of PTMC in females (16.2%) was slightly lower than in males (17.2%), but did not prove to be statistically significant. The youngest patient in our study was 14 years old and the oldest was 85 years old, with a mean age of 54 years. The youngest patient with PTMC was 15 years old and the oldest was 81 years old. The highest incidence of 17.4% was noted in the age group 51-60. Age did not prove to be statistically significantly related to the incidence of PTMC. Even though 47% of all PTMC cases were in the MNG group its incidence (16.4%) did not prove to be statistically significant (χ^2^ = 0.02, p > 0.05). The highest incidence of PTMC (22.7%) was noted in the group of patients with Hashimoto thyroiditis, where there were 72 patients with PTMC (18% of all PTMC cases), and it proved to be highly statistically significant (χ^2^ = 10.80, p < 0.001). The lowest incidence of PTMC (6.6%) was noted in the group of patients with a SHTN where there were only 9 patients with PTMC (2% of all PTMC cases), and this also proved to be highly statistically significant (χ^2^ = 9.96, p < 0.001). There were roughly three times more cases with PTMC in the TT group than in the HT group. The incidence of PTMC was higher in the TT group compared to the HT group (17.7% vs. 13.4%), and was statistically significant (χ^2^ = 7.05, p = 0.008). Table 2 shows the results of the first MLR model we performed where we tested all variables and their relation to PTMC. According to ULR, Hashimoto thyroiditis (OR 1.61, 95% CI 1.21-2.15, p < 0.001), SHTN (OR 0.35, 95% CI 0.18-0.69, p < 0.003) and extent of surgery (OR 1.39, 95% CI 1.09-1.77, p < 0.008) were statistically significantly associated with PTMC. According to our first MLR model, we found that an independent positive predictor of PTMC was Hashimoto thyroiditis (OR 1.54, 95% CI 1.15-2.05, p < 0.003), while a SHTN proved to be a independent negative predictor of PTMC (OR 0.43, 95% CI 0.21-0.87, p < 0.019). Table 3 shows the results of the second MLR model we performed where we tested all variables, including PTMC and their relation to the extent of operation. According to ULR, the extent of operation was statistically significantly associated with all variables, except sex, age and MNG. The results of this MLR showed that each type of BTD is highly statistically significantly related to the extent of surgery performed, except for MNG. Furthermore, the extent of surgery was highly statistically significantly related to the presence of PTMC, proving that the extent of surgery is an independent positive predictor of PTMC (OR 1.45, 95% CI 1.10-1.92, p < 0.009). Table 4 shows the results of the third MLR model we performed where we tested each type of BTD and its relation to PTMC, adjusted for sex, age and extent of surgery. We can clearly observe that an independent positive predictor for PTMC in relation to the type of BTD, is Hashimoto thyroiditis (OR 1.61, 95% CI 1.20-2.15, p = 0.001), whereas Graves disease (OR 0.72, 95% CI 0.53-0.99, p = 0.041) and SHTN (OR 0.40, 95% CI 0.20-0.81, p = 0.011) are independent negative predictors of PTMC. In other words, we can expect a higher incidence of PTMC in patients with Hashimoto thyroiditis, while in patients with Graves’s disease and a SHTN we can expect a lower incidence of PTMC. The results for Plummer's disease came close to statistical significance (OR 0.70, 95% CI 0.49-1.02, p = 0.06) following a similar pattern as for other hyperthyroid diseases.

**Table 1:** **Incidence of PTMC** ^**a**^ **in relation to sex, age, BTD** ^**b**^ **and extent of surgery**

	All cases		Cases with PTMC		Cases without PTMC		Pearson chi-square	
	N	%	N	%	N	%	Value	p
**Sex**								
Males	338	13.7	58	17.2	280	82.8	.191	.692
Females	2128	86.3	345	16.2	1783	83.8		
**Age**								
<20	29	1.2	4	13.8	25	86.2	3.920	.687
21-30	173	7.0	20	11.6	153	88.4		
31-40	353	14.3	56	15.9	297	84.1		
41-50	489	19.8	84	17.2	405	82.8		
51-60	684	27.7	119	17.4	565	82.6		
61-70	545	22.1	88	16.2	457	83.8		
70+	193	7.8	32	16.6	161	83.4		
**BTD**								
MNG^c^	1143	46.4	188	16.4	955	83.6	.017	.913
Hashimoto	317	12.9	72	22.7	245	77.3	10.799	**.001**
Graves	457	18.5	65	14.2	392	85.8	1.842	.183
Plummer's	278	11.3	38	13.7	240	86.3	1.638	.228
SHTN^d^	136	5.5	9	6.6	127	93.4	9.956	**.001**
Benign tumour	875	35.5	142	16.2	733	83.8	.013	.955
**Extent of surgery**								
HT group^e^	762	30.9	102	13.4	660	86.6	7.050	**.008**
TT group^f^	1704	69.1	301	17.7	1403	82.3		
**Total**	2466	100	403	16.3	2063	83.7	N/A	

^a^Papillary thyroid microcarcinoma; ^b^Benign thyroid disease; ^c^MNG Multinodular goitre; ^d^SHTN Solitary hyperfunctional thyroid nodule; ^e^Less than total or near-total thyroidectomy; ^f^Total or near-total thyroidectomy.

**Table 2:** **Univariate and multivariate logistic regression of sex, age, type of BTD** ^**a**^ **and extent of surgery in relation to PTMC** ^**b**^

Variable	OR^c^	95% CI^d^	p^e^
**Univariate logistic regression analysis**			
Sex	.934	.688-1.268	.662
Age	1.025	.965-1.088	.421
MNG^f^	1.015	.819-1.257	.895
Hashimoto	1.614	1.211-2.152	**.001**
Graves	.820	.615-1.093	.175
Plummer's	.791	.552-1.134	.202
SHTN^g^	.348	.176-.691	**.003**
Benign tumour	.987	.790-1.234	.987
Extent of surgery	1.388	1.089-1.770	**.008**
**Multivariate logistic regression analysis** (p < 0.10)			
Hashimoto	1.539	1.153-2.054	**.003**
SHTN	.428	.211-.868	**.019**

^a^Benign thyroid disease; ^b^Papillary thyroid microcarcinoma; ^c^Odds ratio; ^d^Confidence interval; ^e^Statistical significance; ^f^Multinodular goitre; ^g^Solitary hyperfunctional thyroid nodule.

**Table 3:** **Univariate and multivariate logistic regression of sex, age, type of BTD** ^**a**^ **and PTMC** ^**b**^ **in relation to extent of operation**

Variable	OR^c^	95% CI^d^	p^e^
**Univariate logistic regression analysis**			
Sex	1.255	.986-1.598	.065
Age	1.042	.933-1.093	.095
MNG^f^	.986	.831-1.171	.874
Hashimoto	1.327	1.015-1.733	**.038**
Graves	15.255	9.319-24-972	.**000**
Plummer's	5.947	3.813-9.274	**.000**
SHTN^g^	.033	.018-.062	**.000**
Benign tumour	.119	.098-.144	**.000**
PTMC	1.388	1.089-1.770	**.008**
**Multivariate logistic regression analysis** (p < 0.1)			
Hashimoto	1.773	1.320-2.382	**.000**
Graves	10.844	6.499-18.093	**.000**
Plummer's	5.191	3.261-8.264	**.000**
SHTN	.103	.054-.197	**.000**
Benign tumour	.216	.175-.266	**.000**
PTMC	1.452	1.098-1.922	**.009**

^a^Benign thyroid disease; ^b^Papillary thyroid microcarcinoma; ^c^Odds ratio; ^d^Confidence interval; ^e^Statistical significance; ^f^Multinodular goitre; ^g^Solitary hyperfunctional thyroid nodule.

**Table 4:** **Multivariate logistic regression of each type of BTD** ^**a**^ **in relation to PTMC** ^**b**^ **adjusted for age, sex and extent of operation**

Variable	OR^c^	95% CI^d^	p^e^
MNG^f^	1.004	.961-1.085	.974
Hashimoto	1.606	1.200-2.148	**.001**
Graves	.724	.532-.986	**.041**
Plummer's	.704	.487-1.019	.063
SHTN^g^	.402	.199-.813	**.011**
Benign tumour	1.176	.915-1.511	.205

^a^Benign thyroid disease; ^b^Papillary thyroid microcarcinoma; ^c^Odds ratio; ^d^Confidence interval; ^e^Statistical significance; ^f^Multinodular goitre; ^g^Solitary hyperfunctional thyroid nodule.

## Discussion

The prevalence of PTMC in autopsy series ranges from 1.7 to 35.6% [10-13]. In clinical series, the incidence of PTMC in patients operated for BTD is much lower, and ranges from 3% to 17% [14-16]. Our present study revealed a higher incidence of PTMC compared to our previous study of PTMC incidence [17]. First of all, a possible reason for this finding is related to a more radical approach to BTD surgery we advocate now, which was not the case at the time of our previous study. Compared to the time of our previous study, now BTD patients undergo TT twice as often. This has proved to be highly statistically significant in another of our studies. In that study, we compared two periods, a decade apart, and found that in the first period, only a third of BTD patients had a TT; while in the second period nearly 80% of BTD patients, had a TT [18]. Secondly, such an increase of PTMC could be the result of the increasing number of routine histological sections performed on the thyroid gland over the past decade; thus leading to an increased detection of smaller PTMC, that would not have been revealed in past histopathological examinations. Even though such an explanation is proposed by other authors [19]; we do not believe that this could fully explain the noted increase of incidence, especially since our pathohistological protocols have not changed much in the past decade.

In our study we did not find sex to be an independent predictor for PTMC, although there was a 1% higher incidence among male patients (17.2% vs. 16.2%). Even though the male sex is considered a prognostic factor and is independently related to the clinical course of PTMC and even overall survival; it has not been found to be an independent predictor for PTMC [20]. On the contrary, gender is a well known risk factor for PTC [21,22]. These findings imply that PTMC should be treated as a different entity compared to PTC after surgery. In this line, in our study there were approximately six times more females with PTMC than males, but there were also nearly six times more female patients. Noguchi et al. find a even higher female-to-male PTMC ratio in their study (9:1) [8]. Roti et al. agree that sex is not a independent predictor for PTMC and explain the higher incidence of PTMC in women being the result of a higher incidence of BTD in women; thus women are more frequently exposed to diagnostic and therapeutic procedures [23]. Furthermore, autopsy studies decades apart show that gender is not a risk factor for PTMC [11,12].

Similar to our findings for sex, we did not find age to be a independent predictor for PTMC or to be related to PTMC. Again we find evidence that PTMC shows different clinical characteristics compared to PTC. We did find a generally higher incidence of PTMC in older age groups with the peak being in the 51-60 age group, but without statistical significance. More than half of PTMC was in the age range 41-60, but this age range also comprised nearly half of all patients included in the study. Some authors find that age is related to PTMC as a prognostic factor, while others don't, but it is not considered a independent predictor [8,24,25]. Even though certain authors consider age as an independent predictor of PTMC, for patients older than 45 years, autopsy studies do not show such a pattern [13,26].

The incidence of PTMC in MNG is generally high, and based on previous studies, varies significantly ranging from 7 to 17% [17,27-31]. Even though our previous study found that the highest incidence of PTMC was in the MNG group (13.4%), and this study showed an even higher incidence (16.4%), we did not find it to be statistically significant [17]. There is a general consensus that a higher rate of TC is found in goitres with a solitary nodule than in MNG, but numerous studies have demonstrated that in fact there is no difference in the incidence of TC in these two entities [32]. Our study demonstrated that this can be applied to PTMC, also. In the group with benign tumours there was also a high incidence of PTMC, but it did not differ much from the MNG group and was in fact lower (16.2% vs. 16.4%). This coincides with the results of the study of Mihailescu and Schneider, who find that the likelihood of TC is increased if more than one nodule is present in patients at high risk of TC [33]. On the contrary, Barroeta et al. find that TC risk decreases with three or more nodules [34]. Taking this into account, and the results of our study where we did not find a statistical association between PTMC and MNG or benign tumours, we can conclude that these BTDs cannot be considered as independent predictors for PTMC.

Our study showed that Hashimoto disease is an independent positive predictor for PTMC. Previous studies suggest that Hashimoto thyroiditis patients have a higher risk of TC, mainly as a result of chronic inflammation [35-37]. Likewise, Kim et al. find that it is significantly related to PTMC and its clinical course [38]. Fiore et al. in their study of roughly 10,000 patients demonstrated that TSH levels are higher in patients with PTC and that the prevalence increases with TSH, being the highest in patients with serum TSH in the upper limit of the normal range [39]. During the natural course of Hashimoto thyroiditis disease, we can reasonably presume that these patients were exposed to higher levels of TSH, in a certain time frame, and that that this could have in fact be the reason for a higher incidence of PTMC among these patients. Kim and Park give further evidence for this hypothesis in their study that found a similar correlation between the sole level of TSH and TC; even in patients without thyroid autoimmune diseases [40]. In contrast, Sugitani et al. do not find a positive correlation between TSH and PTMC [41]. Probably, the most likely cause for a higher incidence of PTMC in Hashimoto thyroiditis disease is dual; as the result of chronic inflammation and the prolonged influence of a higher level of TSH. We should also consider that patients with high TSH levels, who go untreated for a long period of time, will develop thyroid enlargement and will eventually undergo surgery because of mechanical compression and thus the percentage of such patients would be higher and could artificially increase the rate of incidentally discovered PTMC.

On the contrary, long-term suppressed levels of TSH, as in patients with hyperthyroidism, would imply a lower incidence of TC. This proved to be true in our study where hyperthyroid diseases were found to be independent negative predictors of PTMC. There was a highly significant reduced risk of PTMC in patients with a SHTN or Graves disease (OR 0.40 and 0.72, respectively), and close to significant (p = 0.063) reduced risk (OR 0.70) in patients with Plummer's disease. It seems that a low level of TSH, surpasses the effect of chronic inflammation, as in Graves diseases. Chigot et al. in their study of 861 patients with hyperthyroidism conclude that hyperthyroidism does not play a causal role in the development of TC [42]. A 3% incidence of TC has been reported historically for Plummer's disease; but in a more recent ten-year study comprising 2500 patients an incidence of 18% was found, suggesting that this rate has been underestimated [43]. Our study, with nearly the same number of patients, found a lower incidence (13.7%) for Plummer's disease, but a higher incidence for SHTN (4.5% vs. 6.6%).

In our present study, the incidence of PTMC in BTD is statistically connected with the extent of surgery and we found a threefold increase in the incidence of PTMC in the TT group compared to the HT group. According to our study, there is a higher risk for finding PTMC in patients that undergo TT (OR 1.45). The extent of surgery for BTD is still questionable, and varies from TT, to HT. [44,45] At our Centre we advocate TT, whenever possible, for patients with BTD, except for SHTNs and unilateral benign tumours, since it provides better control of the disease; and as a method of secondary prevention of reoperations that carry a much higher risk of complications. Feroci F et al. in their meta-analysis show that TT offers a better chance of cure of hyperthyroidism than HT and can be accomplished safely with only a small increase in temporary and permanent hypoparathyroidism [46]. Yoldas et al. in their study of 748 patients with MNG, find a high recurrence rate of 29% among their patients who underwent HT; and therefore recommend that HT should be discontinued and TT should be preferred, while keeping in mind the probability of a higher risk of hypoparathyroidism [47]. To reduce the risk of postoperative permanent hypocalcaemia we perform HT, such as the Dunhill operation, as a coerced surgical procedure, for those patients in which we did not reliably identify the vascularisation of the parathyroid glands on the first side of the operated thyroid gland. Furthermore, according to our investigation, we agree with Pisello et al. that TT for patients with BTD is recommended, in the light of a not so insignificant incidence of PTMC in BTD [48]. Patients with incidental PTMC that underwent TT for BTD are easier to follow up, since we can then use thyroglobulin and thyroglobulin antibodies as tumour markers. Moreover, it is easier to adjust the right dose of thyroid hormone replacement therapy for patients with BTD with or without PTMC that underwent TT. Thus these patients require less frequent controls. Finally, mortality, although low, has been reported for PTMC [49]. Naturally, this approach can only be utilized in high-volume endocrine surgery units, where thyroid surgery can be performed safely with minimal morbidity. In such an environment the extent of surgery has no impairment on the quality of life of these patients; regardless of the extent of surgery [50]. It is important to note that the indications for surgery of these patients was the patients' BTD, and not PTMC. We agree with Ito et al. that most low-risk PTMC lacking aggressive features are harmless, and immediate surgery for all of them is definitely an overtreatment [25].

Thyroid nodules are a very common clinical finding, with an estimated prevalence, on the basis of palpation of 3-7%, and on the basis of ultrasound examination of 20-76% [51]. If age and results of autopsy studies are also added in to the equation, it can be said that 50% of 60-year-old persons have thyroid nodules [52]. Acknowledging this fact, the clinician finds himself suddenly in a most awkward predicament. Having detected such a vast number of patients with thyroid lesions, we are in a quandary as to what to do with them. This manuscript was born out of the necessity to find additional criteria that can be implemented in everyday clinical practice and that would help us in providing optimal treatment for such a large number of patients. The first clinical dilemma that arises is how to select, from such a vast number of patients with thyroid lesions, patients that require surgery and secondly, what is the individual appropriate extent of surgery. Fortunately, these are usually benign lesions, but nonetheless we have to evaluate which nodules are suspicious of malignancy. Taking into account that the results of FNAB are not always reliable, especially in patients with MNG with lots of nodules and in patients with nodules smaller than 10mm, it is particularly difficult to establish a sound diagnosis for small non-palpable nodules that have been verified by ultrasound only [53,54]. It is important to note that non-palpable nodules have the same potential for TC as palpable nodules of the same size [55]. Furthermore, the risk of TC is nearly the same in incidentally discovered nodules smaller than 10mm as it is in clinically evident nodules greater than 10mm [56]. Finally, intraoperative macroscopic identification of PTMC is not always possible considering its size; and especially since many endocrine surgery units have stopped routinely using frozen section biopsy, which is considered costly and unnecessary [57].

In this study we determined the incidence of PTMC in BTD and identified potential independent predictors for PTMC. There have not been many studies that examined independent predictors and the incidence of PTMC in BTD with as many patients as in our study. Naturally, it would be useful if this study could be repeated on a higher number of patients, and preferably as a multicentric study that would include patients from multiple high volume endocrine surgery units. Furthermore, additional independent predictors for PTMC could be identified; which is the aim of our other ongoing study. Recognizing potential independent predictors for PTMC would help us in selecting patients for operative treatment and choosing the optimal extent of surgery for their disease.

## Conclusion

Sex and age are not independent predictors for PTMC. Independent positive predictors for PTMC are Hashimoto thyroiditis and a greater extent of surgery. Independent negative predictors for PTMC are a SHTN and Graves disease.
